# Influence of the Inclusion of Propylene Carbonate Electrolyte Solvent on the Microstructure and Thermal and Mechanical Stability of Poly(l-lactic acid) and Poly(vinylidene fluoride-*co*-hexafluoropropylene) Battery Separator Membranes

**DOI:** 10.1021/acs.jpcc.3c02514

**Published:** 2023-05-31

**Authors:** Luis Amaro Martins, Laura Teruel Biosca, José Antonio Gómez-Tejedor, J. P. Serra, Daniela M. Correia, Carlos M. Costa, Senentxu Lanceros-Méndez, José Luis Gómez Ribelles, Isabel Tort-Ausina

**Affiliations:** † Centre for Biomaterials and Tissue Engineering, CBIT, Universitat Politècnica de València, 46022 Valencia, Spain; ‡ Physics Centre of Minho and Porto Universities (CF-UM-UP), University of Minho, 4710-057 Braga, Portugal; § Laboratory of Physics for Materials and Emergent Technologies, LapMET, University of Minho, 4710-057 Braga, Portugal; ∥ Centre of Chemistry, University of Minho, 4710-057 Braga, Portugal; ⊥ BCMaterials, Basque Center for Materials, Applications and Nanostructures, UPV/EHU Science Park, 48940 Leioa, Spain; # Ikerbasque, Basque Foundation for Science, 48009 Bilbao, Spain; ∇ Biomedical Research Networking Center on Bioengineering, Biomaterials and Nanomedicine (CIBER-BBN), 46022 Valencia, Spain

## Abstract

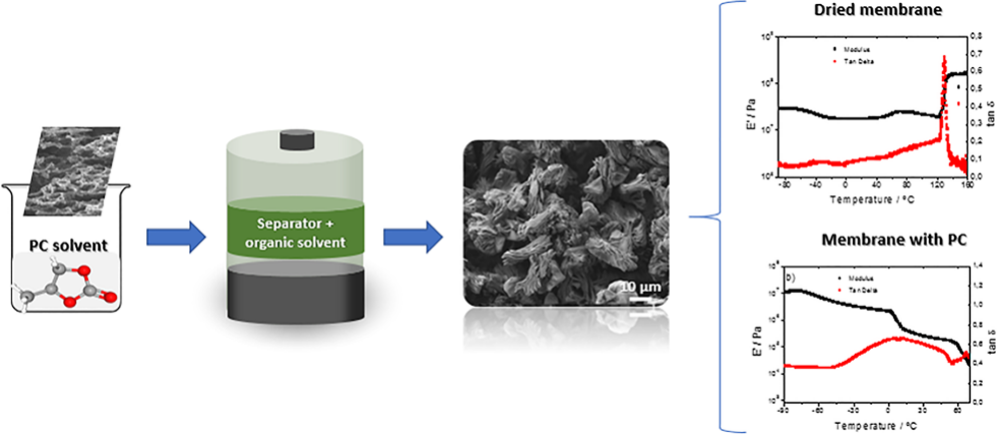

The influence of the inclusion of the organic solvent propylene carbonate (PC) in microporous membranes based on poly(l-lactic acid) (PLLA) and poly(vinylidene fluoride-*co*-hexafluoropropylene) P(VDF-HFP) has been studied based on its relevance for the application of those separator membranes in lithium-ion batteries. The membranes have been produced through solvent casting and characterized with respect to the swelling ratio originated by the uptake of the organic solvent. The organic solvent uptake affects the porous microstructure and crystalline phase of both membrane types. The organic solvent uptake amount affects the crystal size of the membranes as a consequence of the interaction between the solvent and the polymer, since the presence of the solvent modifies the melting process of the polymer crystals due to a freezing temperature depression effect. It is also shown that the organic solvent partially penetrates into the amorphous phase of the polymer, leading to a mechanical plasticizing effect. Thus, the interaction between the organic solvent and the porous membrane is essential to properly tailor membrane properties, which in turn will affect lithium-ion battery performance.

## 1 Introduction

One of the most efficient technologies for energy storage, highly applied in mobile electronic applications such as smartphones, computers, or wearable gadgets, and being increasingly applied in the transportation area through the implementation of the electric vehicle, is lithium-ion batteries.^1,2^ Those electrochemical cells convert chemical energy into electric energy^3−5^ and are characterized by being light and cheap and having high energy density (>210 Wh·kg^–1^), low charge loss, no memory effect, and high number of charge/discharge cycles when compared to other battery systems such as NiCd (nickel–cadmium) or NiMH (nickel–metal-hydride).

The basic constituents of Li-ion batteries are the anode, the cathode, and the separator membrane, the latter being the medium for the transfer of ionic charges.^6−8^ The separator is generally a porous polymer matrix soaked by an electrolyte solution, i.e., a liquid electrolyte where salts are dissolved in a solvent, water, or organic molecules.^9^ The solvents present in the electrolyte solution must meet specific requirements for battery applications, which are contradictory in some cases.^10^ The characteristics of an ideal solvent are a high dielectric constant to dissolve large salt concentrations, low viscosity for improving ion transport, being inert to the different cell components, and the ability to remain in the liquid state in a wide temperature range.^10^ The nonaqueous solvents most used in electrolyte solutions for battery applications belong to the classes of organic esters and ethers:^11^ ethylene carbonate (EC), propylene carbonate (PC), dimethyl carbonate (DMC), and diethyl carbonate (DEC).^12−15^ The main differences between the aforementioned solvents are the melting temperature (from −48.8 to 36.4 °C), viscosity (0.50–2.53 cP), and dielectric constant (2.8–90).^10^ One issue that deserves further and deeper attention is that the aforementioned solvents can introduce modifications in the polymer membrane, severely affecting battery performance.^16^ Different polymers have been used for the development of Li-ion battery separators, one of the widely used polymers being poly(vinylidene fluoride), PVDF, and its copolymers poly(vinylidene fluoride-trifluoroethylene), P(VDF-TrFE),^17^ and poly(vinylidene fluoride-*co*-hexafluoropropylene), P(VDF-HFP).^18^ This is due to their good mechanical properties, wetting by the liquid electrolyte, chemically inertness, good contact between electrode and electrolyte, and being stable in cathodic environment due to the low value of the HOMO band.^19^ Different studies report on the influence of organic solvents in PVDF membranes. Thus, PVDF membranes have been immersed in DEC, PC, and γ-butyrolactone (GBL) containing lithium tetrafluoroborate (LiBF_4_), leading to the formation of thermoreversible gels and PVDF chain conformation transitions after the addition of lithium salts to the solvent solution.^20^ The swelling phenomena in dense PVDF membranes has been studied also with liquid organic solvents (EC, DMC, DEC) and lithium salts (LiPF_6_), the electrolyte solution leading to variations of the thermal and mechanical properties of the PVDF membrane through modifications of the microstructure and the crystallinity.^16^

The biopolymer poly(l-lactic acid) (PLLA) has been also shown to be suitable for battery applications in an approach to reduce the environmental impact of battery separators through the replacement of traditional synthetic polymers by more sustainable ones considering their advantages, such as biodegradability and recyclability, presenting also suitable thermal and mechanical characteristics.^21^ PLLA separators have been produced by solvent casting, establishing a correlation between polymer concentration in solution, membrane morphology, porosity, and battery performance.^21^

Propylene carbonate (PC) is a glass-forming liquid. The liquid cooling shows a melting temperature at −48.8 °C, and a glass-transition temperature, *T*_g_, at about −115 °C.^22−24^

PC has been used for dissolving lithium salts for Li-ion battery applications^25^ and is one of the most used, considering its large liquid temperature range (−48.8 to 242.0 °C) when compared to other solvents.^26^ For battery applications, the liquid electrolyte is introduced into the pores of the polymeric membrane, therefore being important to evaluate the interaction of the solvent with the polymer, in particular with increasing temperature, something that occurs in the charging and discharging processes of the battery, as it can lead to morphological, polymer conformation or crystallinity variations of the separator membrane.

In the present work, the influence of the organic solvent PC without lithium salts has been evaluated in two porous membranes: poly(l-lactic acid), PLLA^21^ and a copolymer of vinylidene fluoride, poly(vinylidene fluoride-*co*-hexafluoropropylene), P(VDF-HFP).^27^ Both materials are piezoelectric polymers and have been proposed as battery separators, with high battery performance.^21,27^ Both types of membranes are produced by dissolving them in a suitable solvent (mixtures of *N*,*N*-dimethylformamide (DMF) and dichloromethane (DCM) in the case of PLLA^21^ and DMF in the case of P(VDF-HFP)^27^). In both cases, solvent evaporation at room temperature allows the development of porous membranes, and the effect of the inclusion of the PC solvent on membranes’ microstructure, crystallinity, and mechanical properties is evaluated.

The melting process of a polymer membrane that retains a solvent in its pores is a process controlled by thermodynamic equilibrium. The temperature at which the melting starts (and thus the onset of the softening of the membrane) depends on the solvent content and can be much lower than the melting temperature of the pure polymer. In this work, we analyze the melting process of two membranes: PLLA and P(VDF-HFP) with PC in their pores, something that, to our knowledge, is not found in the literature. Also, in this work, the polymer membrane is formed by crystallization from the mixture with PC. The amount of PC in the pores is what establishes the thermodynamic equilibrium between the semi-crystalline polymer and the solvent. In the studies regarding the dependence of the polymer melting process on the amount of PC retained in its pores, we chose the maximum PC content, which is the one reached when the sample is allowed to equilibrate with liquid PC, which is 340% in the case of PLLA and 201% in the case of P(VDF-HFP), both measured on a dry basis. We have added several intermediate points by controlling the amount of PC retained in the membrane pores by allowing part of the PC to evaporate from an initially swollen sample to equilibrium.

## 2 Experimental Section

### 2.1 Materials

Poly(vinylidene fluoride-*co*-hexafluoropropylene), P(VDF-HFP) (Kynarflex P(VDF-HFP) 2801-00107), and poly(l-lactic acid) (PLLA) (Purasorb PL18) were supplied by Arkema and Purasorb, respectively.

*N*,*N*-dimethylformamide (DMF), dichloromethane (DCM), and propylene carbonate (PC) were acquired from Merck. All materials and chemicals were used as supplied.

### 2.2 Preparation of Membranes

P(VDF-HFP) and PLLA membranes were prepared by dissolving the polymer powders in DMF and DCM/DMF (70:30 volume ratio) at a 15/85 and 10/90 polymer/solvent weight ratio, respectively. These ratios were selected considering the results obtained in^27^ for P(VDF-HFP) and^21^ for PLLA.

The polymers were dissolved in the respective solvents under magnetic stirring at room temperature. After complete dissolution of the polymers, these solutions were placed in a glass Petri dish for solvent evaporation at room temperature. The final thickness of the membranes is ∼100 μm.

### 2.3 Sample Characterization

The surface and cross-sectional morphologies of the membranes were evaluated with a field emission scanning electron microscope (FESEM; ZEISS Ultra-55) at 30 kV, 500 pA. A platinum conductive layer was first deposited on the samples by magnetron sputtering (Polaron, model SC502).

Differential scanning calorimetry (DSC) was carried out with a DSC Mettler Toledo 823e in the temperature range −145 to 200 °C at 10 °C/min for two scans. A DSC 8000 from PerkinElmer was also used for scans in the melting region (20–200 °C at 20 °C/min) in both cases under flowing nitrogen (N_2_) atmosphere.

Mechanical evaluation was carried out by dynamic mechanical analysis (DMA) with a PerkinElmer DMA 8000 apparatus in compression mode. Samples of approximate dimensions 8 × 5.5 × 0.1 mm^3^ were used. The storage modulus (*E*′) and loss tangent (tan δ) were measured as a function of temperature at a frequency of 1 Hz from −90 to 160 °C at a rate of 3 °C/min.

The porosity of the samples was calculated by weighing. PC was introduced in the samples under vacuum and then kept in immersion for 24 h. Then, the porosity, ϕ, has been obtained after weighing (A&D Instruments, cat. No. GR-200) and applying eq 1(28)
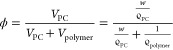
1where *w* is the weight of absorbed PC per gram of polymer and *w* = ρ × *V*. The densities are ρ_PC_ = 1.2 g/cm^3^ for PC,^29^ ρ_PLLA_ = 1.25 g/cm^3^ for PLLA,^30^ and ρ_P(VDF-HFP)_ = 1.78 g/cm^3^ for P(VDF-HFP).^31^ The obtained porosities are: ϕ_PLA_ = 0.78 and ϕ_P(VDF-HFP)_ = ∼0.70.

## 3 Results and Discussion

### 3.1 Sample Morphology and Thermal Analysis

**Figure 1:**
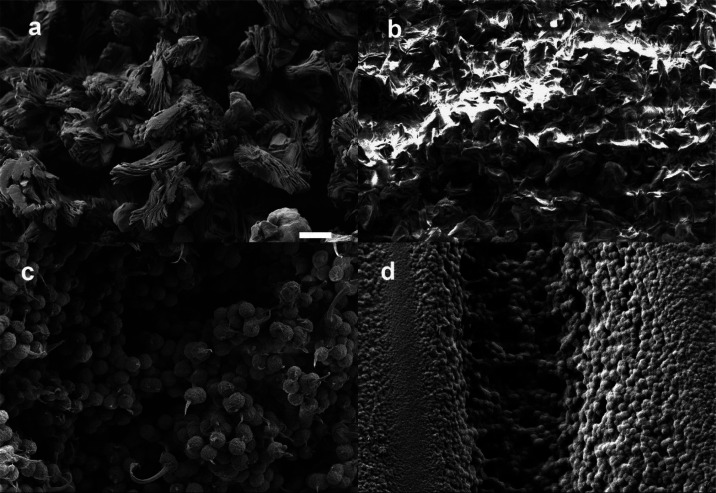
FESEM images of the dried samples of neat PLLA (a) and P(VDF-HFP) (c) membranes; panels (b) and (d) show the microstructure of PLLA and P(VDF-HFP) membranes, respectively, after heating the PC-swollen membrane to polymer fusion and then cooling and vacuum-drying to remove the PC. The scale bar in panel (a) corresponds to 10 μm and is valid for the four pictures.

PLLA was produced by evaporation of the solvent (a mixture of DCM and DMF in a 70:30 volume ratio) at room temperature, resulting in a semi-crystalline material. Crystallization is in the form of aggregates composed of thin sheets, as it can be observed in Figure 1a. This leads to a highly porous structure in which large interconnected irregular macropores with sizes in the order of tens of microns and a microporosity with sizes of the order of one micron are observed. On the contrary, the P(VDF-HFP) copolymer crystallizes in the characteristic spherulitic semi-crystalline microstructure.^27^ Each of the microspheres observed in Figure 1c with diameters of the order of 5 μm represents a spherulite of the copolymer. These leaves, as in the case of PLLA, a macro- and microporous architecture in which the PC will be housed.

The porous architecture of the membranes is very sensitive to the solvent casting process, in particular to the temperature at which solvent evaporation occurs, and it is possible to modulate it by means of heat treatments.^32^ One of the relevant issues to consider is to what extent the PC penetrates into the polymer structure. In principle, it is possible to think that, in addition to occupying the pores, the solvent diffuses into the amorphous phase of the semi-crystalline polymers, which in both membranes is expected to be distributed in the interlamellar regions.^33^ Another possibility is that part of the polymer crystallites dissolves in the PC, as shown in Figure 1b,d.

After heating the PC-swollen membrane to polymer fusion and then cooling and vacuum-drying to remove the PC, the PLLA membranes lose their microporosity, as observed in Figure 1b. Melting the PLLA crystals and re-forming them from the melt in the presence of PC seems to lead to more compact PLLA aggregates; however, it is noteworthy that the macropore structure is largely maintained, even though the sample appears less porous. In this case, the PLLA crystals are formed from the liquid mixture that can be treated as homogeneous at 200 °C. The growth of PLLA crystals causes them to separate from the liquid. The proportion of PLLA and PC in the mixture means that a membrane with a large part of the volume occupied by liquid PC is obtained. The change in the microstructure of the P(VDF-HFP) membranes is even more noteworthy, with crystallization from the melt leading to almost complete collapse of the pore structure (Figure 1d).

**Figure 2:**
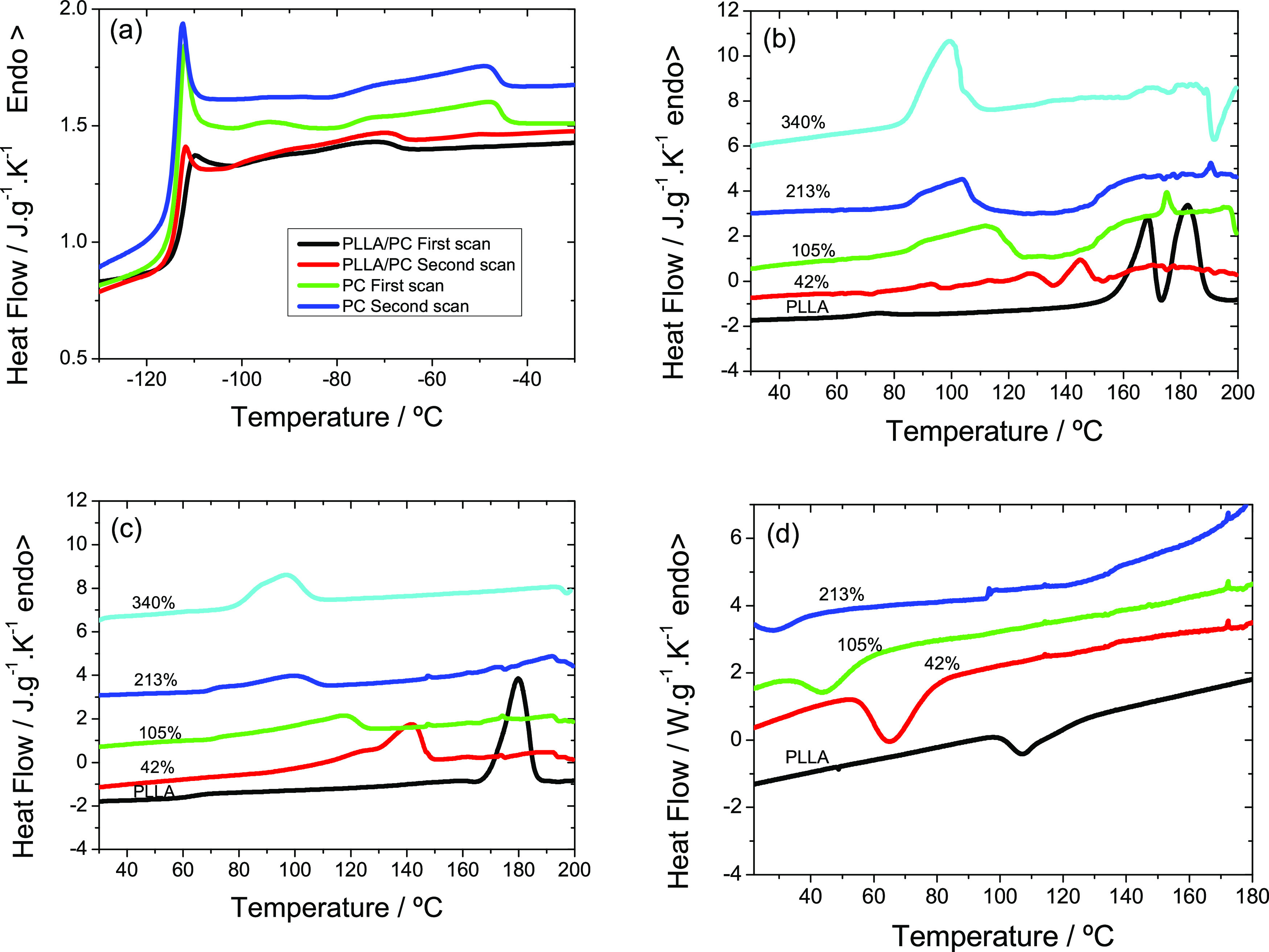
(a) DSC thermograms for PC and for the PLLA membrane containing the maximum amount of PC in the porous structure, *w* = 3.4 g of PC/g of PLLA in the glass-transition region of pure PC. DSC scans of PLLA membranes containing PC: (b) first heating scan, (c) second heating scan. (d) Cooling scan for pure PLLA and different contents of PC (42, 105, 213, and 340% by reference to dry weight of the membrane).

The glass-transition temperature (*T*_g_) of PC is a very sensitive parameter to the mixture between the PC and the amorphous chains of the polymer. In the case of the PLLA membrane, if this mixture occurs, a displacement of the *T*_g_ toward high temperatures should be observed, or a double transition, if a part of the PC penetrates the PLLA and another part remains in the pores of the polymer. Figure 2 shows the low-temperature section of the DSC thermograms of pure PC and the PLLA membrane containing the PC within the pores.

The glass transition of PC in the membrane formed by cooling from the melt is almost identical to that of pure PC. This means that the miscibility of PC with the amorphous chains of PLLA is very low. The PC is segregated from the PLLA phase even more effectively than when the membrane is first produced, and then the PC is introduced.

The glass transition of pure PC is clearly observed in the heating thermogram (Figure 2). The *T*_g_ measured as the midpoint of the *c*_p_ increase in the transition is −114.5 ± 0.5 °C, and the *c*_p_ increase in the transition is Δ*c*_p_ = 0.54 kJ/kg·K. It is observed that in the first heating scan (heat 1) of the membranes containing PC, the transition shifts very slightly toward high temperatures. The *T*_g_ becomes −113 °C, but the increase in *c*_p_ in the transition measured per gram of PC in the sample practically remains constant Δ*c*_p_ = 0.52 kJ/kg·K. It is also verified that the glass transition widens with respect to pure PC. This would be indicative that the solvent penetrates the amorphous phase of PLLA, but to a small extent.^34^

At temperatures above *T*_g_, a broad endothermic process is observed in pure PC, which can be related to the melting of a small crystalline fraction.^35^ A process is also observed in this zone, around −70 °C, in the PC-containing membrane.

In the first heating scan, the glass transition of PLLA is observed between 60 and 80 °C (Figure 2b,c), with a value of *T*_g_ = 69 °C (midpoint of the *c*_p_ increase). The melting process of the PLLA membrane crystals is presented in Figure 2b. Two endothermic peaks appear in a row. The explanation is that the membrane has been formed by crystallizing the PLLA from the solution at room temperature. The size of the crystals formed (the thickness of the lamella) is consequently very small because the crystallization temperature is well below the equilibrium melting temperature. These crystals melt at a very low temperature.^36^ In the thermogram, the melting onset is observed around 150 °C. In the heating scan itself, the molten PLLA chains in that temperature range crystallize again. This crystallization process is reflected in a sudden decrease in the heat flow, that is, a displacement of the thermogram toward the exothermic side. A few degrees higher, the newly formed crystals melt again, now at normal PLLA temperatures. As observed in Figure 2c, the second heating scan already shows a single peak at temperatures close to those of the highest temperature peak of the first heating scan. This is verified due to the fact that the crystals that are melting were formed from the melt during the cooling scan at around 110 °C (Figure 2d). The crystalline fraction of PLLA in the original membrane has been calculated from the first heating thermogram, integrating the peak taking a baseline drawn between 80 °C (after the glass transition, with the amorphous phase in the liquid state and when the melting of the crystals has not yet started) and 200 °C (when all of the polymer is melted). Taking a value of Δ*H*_m_0__ = 93,7 J/g for the melting enthalpy of the 100% crystalline PLLA,^37^ a crystalline fraction of 60% is obtained.

DSC thermograms were also recorded for membranes containing varying amounts of PC. To do that, PC was allowed to evaporate from the membranes swollen at equilibrium for 24 h until the desired weight content of PC was obtained. When the heating scan is carried out in the samples containing PC within the pores, it is observed that regardless of the PC content of the sample, the thermogram starts deviating toward the endothermic side at a temperature of around 85 °C. The endothermic process associated with the melting of the PLLA crystals presents a peak at a temperature that decreases with increasing content of PC. This is a phenomenon associated with cryoscopic descent:^38^ in the presence of a solute (here PC), the melting temperature of PLLA is much lower than that of pure PLLA. At that temperature, once the *T*_g_ of the amorphous phase is exceeded, the solvent quickly penetrates the spherulites, the PLLA crystals melt, and a homogeneous solution is formed. The same effect that has been observed in pure PLLA somehow also occurs with the liquid mixture of PLLA and PC. At a temperature that depends on the PC content, crystallization begins in the heating scan itself, and the thermogram deviates toward the exothermic side. This onset of crystallization occurs at 130 °C when the mixture contains 42% PC, at 115 °C with 105%, 105 °C with 215%, and 100 with 340%. This decrease in the crystallization temperature with increasing solute content in a binary mixture (here, the solute is now the PC) is what predicts the thermodynamic equilibrium,^39^ again associated with the cryoscopic decrease.

When the sample reaches 200 °C, it is a liquid and homogeneous solution of PLLA and PC. Cooling scans show the crystallization peak of PLLA from this liquid mixture, and again the cryoscopic descent is appreciated (Figure 2d). The subsequent second heating scan shows the very broad melting peaks characteristic of two-component mixtures, again the melting temperature being much lower with higher PC content (Figure 2c).

**Figure 3:**
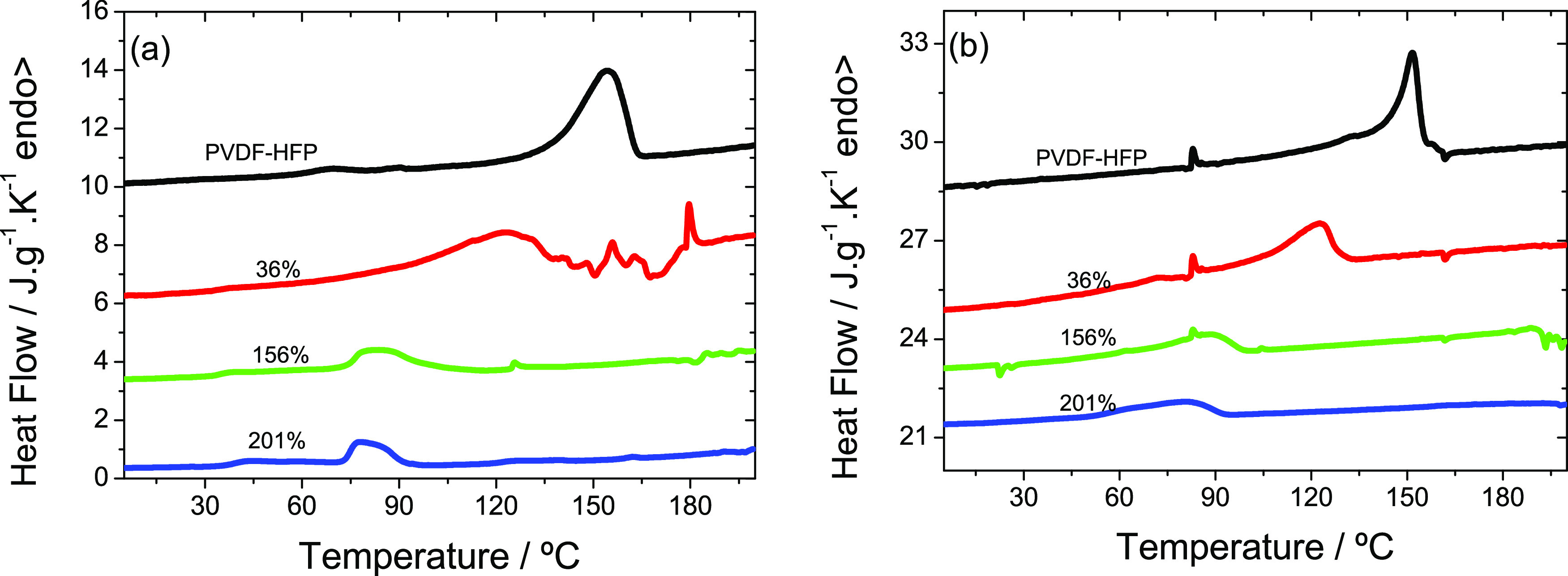
DSC scans of the membranes containing P(VDF-HFP): (a) first heating scan, (b) second heating scan for pure P(VDF-HFP), and different contents of PC (36, 156, and 201%).

The result with the P(VDF-HFP) membrane is completely parallel to what it has been described for the PLLA membrane (Figure 3). The heating thermogram of the original membrane shows a single melting peak whose integration allows to calculate the crystallinity of the sample.

Taking into account that the relative fraction of the β-phase is predominant in these types of membranes^40^ and that the melting enthalpy for a 100% β-PVDF crystalline sample is 104.40 J/g,^41^ a 59% of crystalline fraction has been obtained in this membrane.

The effect of cryoscopic descent in the original membrane, as well as crystallization during the cooling scan and in the second heating scan, is clearly observed in Figure 3.

### 3.2 Mechanical Analysis

**Figure 4:**
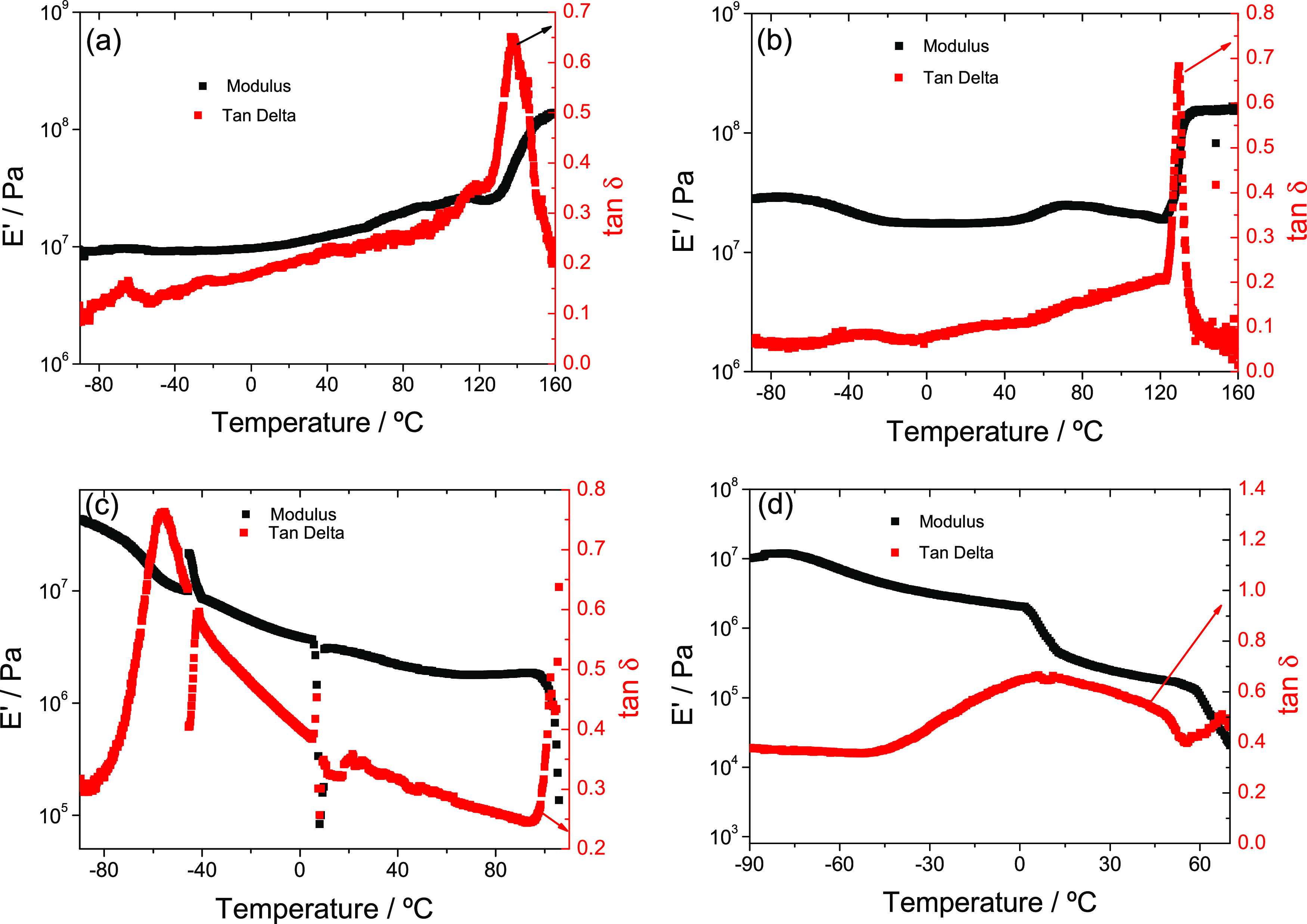
(a, c) Dried PLLA and P(VDF-HFP) membrane measured in compression. (b, d) PLLA and P(VDF-HFP) membrane with PC inside the pores.

The dynamic mechanical properties have been measured in compression mode. In dry PLLA, the sponge collapses as the temperature increases (Figure 4a). Thus, as the pore volume fraction decreases, the elastic modulus increases.

Upon reaching the region of the main relaxation of PLLA (the T_g_ observed in DSC is 69 °C, so α relaxation appears around 100–120 °C, at 1 Hz), the elastic modulus already begins to fall, but when the sample softens, it yields faster to the applied compressive force, collapsing the pore structure and causing the elastic modulus to increase rapidly. At the highest temperatures, a modulus of the order of 10^8^ Pa is obtained, which remains constant as the temperature increases and corresponds to the rubber modulus of nonporous block PLLA.^42^ When the membrane contains the PC inside the pores, the behavior changes completely, as the solvent retains the structure of the PLLA membrane (Figure 4b). The fall in the elastic modulus between −80 and −50 °C is associated with the main relaxation of the PC inside the pores. At temperatures below this transition, the PC is in the glassy state, and the modulus of the assembly is high but decreases continuously after the PC transitions to the liquid state. At room temperature, the modulus is in the order of 3 MPa, which is the modulus of a more or less rigid rubber. The pure PLLA membrane is more rigid (Figure 4a). It is possible that the PC that penetrates the amorphous phase of the PLLA plasticizes it, and the result is a lower elastic modulus. The properties of PLLA with PC are maintained up to 100 °C; then, the melting process previously observed in the DSC thermograms begins, and the module plummets. It is important to notice that this behavior frames the applicability window of the material.

In the case of dry P(VDF-HFP) membranes, the modulus remains more or less constant during the heating process until reaching a temperature of 120 °C, as shown in Figure 4c,d.

As in the case of PLLA, the pores of the sponge begin to collapse, although it seems that in this case, the pore collapse that produces an increase in modulus is compensated by the decrease in modulus with increasing temperature, so that the modulus is maintained approximately constant, with a small oscillation. By increasing the temperature above 120 °C, the structure collapses quickly under the applied compression force, producing a rapid increase in modulus. What is obtained at the highest temperatures is a modulus of the order of 2 × 10^8^ Pa, which remains constant as the temperature further increases, which would be the nonporous block P(VDF-HFP) rubber modulus. When the membrane contains the PC inside the pores, the solvent retains the membrane structure of P(VDF-HFP) (Figure 4c). Again, as in the previous case, the fall in the elastic modulus between −80 and −50 °C is associated with the main relaxation of the PC inside the pores. Another drop in the modulus is observed starting at around 3 °C, and up to room temperature, the modulus of the assembly is below 1 MPa, which is the modulus of soft rubber. The neat P(VDF-HFP) sponge is more rigid (Figure 4c). It is possible that the PC that penetrated the polymer plasticizes the amorphous phase of the P(VDF-HFP), which results in a lower elastic modulus. The properties of P(VDF-HFP) with PC are maintained up to 60 °C; then, the melting process starts, and the modulus drops rapidly (Figure 4d).

Overall, it has been shown that the PC solvent can occupy the porous structure of the membranes. At the same time, the solvent partially penetrates into the amorphous phase of the polymer, with a plasticizing effect that causes the elastic modulus of the membrane with the solvent inside it to drop very significantly compared to the pure polymer membrane. On the other hand, the presence of the solvent modifies the melting process of the polymer crystals due to a freezing temperature depression effect. The melting process starts in the membranes at a temperature that depends on the PC content but can be as low as 70 or 80 °C. The control of these effects is very important since they affect the ionic conductivity value and, consequently, the battery performance.

## 4 Conclusions

Porous membranes are an essential component of current lithium-ion batteries. For lithium-ion batteries, the porous membrane is soaked in an electrolyte solution (organic solvent with lithium salts) and serves as a medium for the transfer of ions between the two electrodes. In this work, the effect of introducing the organic solvent propylene carbonate (PC) in microporous poly(l-lactic acid) (PLLA) and poly(vinylidene fluoride-*co*-hexafluoropropylene) P(VDF-HFP) membranes has been evaluated, as it is an essential factor determining the performance of battery separators.

The PC solvent content affects the microstructure of the separator and the crystallization kinetics. The crystal size of both polymers is influenced by the presence of the organic solvent in the membranes, which is explained through the thermodynamic equilibrium and the cryoscopic descent. Regarding the mechanical properties, the elastic modulus decreases from 10^7^ to 10^6^ MPa with the inclusion of the organic solvent in all membranes due to the plasticizing effect of the PC solvent penetrating the amorphous phase of the polymer.

It is thus concluded that the proper selection of the organic solvent and solvent content present in the electrolyte solution is essential for optimizing the performance of the battery separator, including its thermal and mechanical stability.
